# Clinical characterization of patients with HLA-B27-associated uveitis and evaluation of the impact of systemic treatment on the recurrence rate: a cross-sectional study

**DOI:** 10.1186/s12348-023-00352-3

**Published:** 2023-08-30

**Authors:** Juan Sebastián Pineda-Sierra, Carlos Cifuentes-González, William Rojas-Carabali, Paula Tatiana Muñoz-Vargas, Alejandro Henao-Posada, Alejandra de-la-Torre

**Affiliations:** 1 Neuroscience (NEUROS) Research Group, Institute of Translational Medicine (IMT), Neurovitae Research Center, Escuela de Medicina Y Ciencias de La Salud, Universidad del Rosario, Carrera 24 # 63C – 69, Bogotá, Colombia; 2 Ophthalmology Interest Group, Neuroscience (NEUROS) Research Group, Institute of Translational Medicine (IMT), Neurovitae Research Center, Escuela de Medicina Y Ciencias de La Salud, Universidad del Rosario, Carrera 24 # 63C – 69, Bogotá, Colombia

**Keywords:** Biologic therapy, Complications, HLA-B27-associated uveitis, Ocular inflammation, Recurrences

## Abstract

**Introduction:**

Despite HLA-B27-associated uveitis is one of the most frequent etiologies of uveitis worldwide, there are scarce studies on the clinical spectrum of this disease and the implications of therapeutic strategies used in the Latin-American population, with none conducted in Colombia. Thus, this study aimed to describe the clinical characteristics of a cohort of patients with positive HLA-B27-associated uveitis in Colombia and evaluate the impact of systemic treatment on the recurrence rate.

**Methods:**

We retrospectively reviewed 490 clinical charts of patients with uveitis, searching for those with positive HLA-B27-associated uveitis over eight years in a referral center in Bogotá, Colombia. We used descriptive statistics to summarize demographic and clinical characteristics and conducted a Chi-square test, Fisher Exact test, Spearman correlation, and Mann–Whitney test to assess associations between treatment strategies and the recurrences rate.

**Results:**

We analyzed 39 patients (59% females) with positive HLA-B27-associated uveitis, with a median age at the first consultation of 44.5 years (Range: 2–80) and a mean follow-up time of 86.4 weeks (1.65 years). Most patients had unilateral uveitis (53.8%) and an anterior anatomical diagnosis (76.6%); two had anterior chamber fibrinous reaction, and only one had hypopyon. Most patients did not show associated systemic symptoms (66.7%). Topical corticosteroids, NSAIDs, methotrexate, mydriatics, and adalimumab were the most used treatments. The most common complications included cataracts, posterior synechiae, and macular edema. We identified that the rate of recurrences decreases over time (*r* = -0.6361, *P* = 0.002571), and this decrease seems to be associated with the initiation of disease-modifying antirheumatic drugs (DMARDs) in chronic and recurrent cases.

**Conclusion:**

The clinical spectrum of HLA-B27-associated uveitis in Colombian patients is distinct from other latitudes. Notably, we found a female predominance, older age at presentation, higher frequency of bilateral and vitreous involvement, and lower frequency of concomitant systemic diseases. Additionally, our results suggest that DMARDs such as methotrexate and biologic agents are good therapeutic options to avoid recurrences in chronic and recurrent cases.

## Introduction

Uveitis is the inflammation of the middle and vascularized eye layer named the uvea. It can be caused by infectious and non-infectious etiologies and is responsible for 2–10% of blindness worldwide [1]. Among these etiologies, Human Leukocyte Antigen B27 (HLA-B27)-associated uveitis emerges as one of the most frequent causes of uveitis [2, 3].

Typically, HLA-B27-associated uveitis is recognized as anterior uveitis [4], the most common anatomical subtype of uveitis worldwide and the third most common in Colombia, representing 23% of all cases globally [5, 6]. This is due to the high prevalence of the HLA-B27 allele among patients with uveitis, described as high as 67% [7]. However, data between studies are heterogeneous and highly influenced by the prevalence of the allele in the population studied, which is 8% among Caucasians and 1–5% in Africa, Asia, and Arabia [7].

As for management, HLA-B27-associated uveitis is mainly treated with topical corticosteroids and mydriatic agents [8, 9]. Nevertheless, due to the high proportion of patients that show recurrences, other therapeutic approaches must be implemented to reduce them, alongside complications and cumulative damage, as recommended by the Fundamentals of Care for Uveitis Initiative [10].

Currently, only one study describes the clinical spectrum of this type of uveitis in Colombia; however, its analysis was limited to patients with a clinical diagnosis of seronegative spondyloarthropathies, and only 29.1% of its sample underwent HLA-B27 testing [11]. No studies describe the implications of treatment strategies in the Colombian population. Additionally, there is scarce evidence of recurrence prevention with systemic therapies other than sulfasalazine and TNFα inhibitors, with no studies conducted in Latin America [9, 12, 13]. Therefore, this study aims to describe the clinical characteristics and therapeutic approaches used in a group of patients diagnosed with HLA-B27-associated uveitis in Bogotá, Colombia, and evaluate the impact of systemic treatment on the recurrence rate.

## Methods

### Design

We conducted a cross-sectional study in patients with HLA-B27-associated uveitis who consulted a uveitis referral center between 2013 and 2021 in Bogotá, Colombia. We followed the STROBE guidelines [14]. This study adheres to the tenets for human research established by the Helsinki Declaration, the Belmont Report, and Colombian Resolution 008430 of 1993. Additionally, this study was approved by the ethics committee of Universidad del Rosario.

### Population

We reviewed 490 electronic clinical records of patients with uveitis from 2013 to 2021 and included patients diagnosed with uveitis and positive HLA-B27 test of any age or sex.

The same uveitis specialist examined all patients. They received detailed eye examinations, physical examinations, and laboratory workup for infectious or non-infectious etiologies, including complete blood count, erythrocyte sedimentation rate, C-reactive protein (CRP), urine analysis, venereal disease research laboratory (VDRL), fluorescent treponemal antibody absorption (FTA-ABS), Mantoux test, and chest radiography. Based on specific clinical findings, antibodies profile, HLA-B27, specific infectious tests, and diagnostic imaging techniques were ordered. All patients were treated in an interdisciplinary way. They were evaluated by infectious disease specialists or rheumatologists when needed.

### Data collection

We elaborated and validated a database in Microsoft Excel (Microsoft Corp., Redmond, WA, USA). Variables included in the database were: Age at diagnosis and age at first episode, sex, clinical diagnosis according to the SUN classification system [5], laterality, clinical course, duration, best-corrected visual acuity (BCVA) at the initial visit and the worst BCVA registered during the follow up, since last visual acuity should not be reported in a series of patients with variable follow-up according to SUN guidelines [5], etiological diagnosis, slit-lamp examination findings, the number of recurrences, complications, time of follow-up, and all treatments received. We collected the data from both eyes, but we selected the worst eye to report the complications and other endpoints (i.e.: BCVA) in bilateral cases, and if both were eligible, they were chosen randomly [15]. Visual acuity of count fingers at 1 mt was converted to 2 LogMAR, hand motion to 2.3, light perception to 2.7, and no light perception to 3.0 [16, 17].

A recurrence was defined as an episode of ocular inflammation after periods of more than three months of inactivity. The recurrence rate was calculated by dividing the number of recurrences over the time of follow-up (in years) and was reported as recurrences per year.

Topical prednisolone and topical cycloplegic drops were the mainstays of treatment for anterior uveitis. Subconjunctival injections of prednisolone were given when intense anterior segment inflammation did not respond to topical therapy. Posterior segment inflammation, cystoid macular edema, and ocular hypotony required the use of oral prednisolone. Sub-Tenon injection of triamcinolone acetonide was used in some cases of cystoid macular edema at the specialist's discretion. Methotrexate and other conventional immunosuppressants were used in refractory cases and patients with active systemic disease. Biologic agents were prescribed mainly for patients with active systemic illness and in refractory or recurrent cases despite the use of steroid-sparing agents. All the DMARDs (immunosuppressants and biologics) were grouped into the same category for some statistical analysis.

### Statistical analysis

First, we used descriptive statistics to summarize demographic and clinical characteristics. We reported mean, standard deviations (SD), and range for continuous variables when normally distributed. In any other case, median and interquartile ranges were used. We reported frequencies and percentages for the categorical variables. Then, a sub-analysis to evaluate the impact of the treatment on the recurrences rate was done in those patients with chronic and recurrent course and a follow-up ≥ 6 months (24 weeks) (*n* = 18). We performed a Spearman correlation between continuous variables and a Mann–Whitney test to compare means between two groups. Additionally, bivariate analyses were performed using the Chi-square test or Fisher's exact test (when applicable). *P* values less than 0.05 were considered statistically significant. Data analysis was performed with the jamovi project (2021). jamovi. (Version 1.6) [Computer Software]. (Retrieved from https://www.jamovi.org).

## Results

We found 39 patients with positive HLA-B27 allele typing, representing 7.95% of all patients seen between January 2013 and December 2021. Females (*n* = 23) were the most frequently affected, representing 59% of cases, the mean age at consultation was 44.5 years with a range of 5–80, the mean age at first episode was 41.8 years, and the mean follow-up time was 86.4 weeks (Range:0–341) with 76.9% (*n* = 30) of patients followed for more than a year. Table 1 summarizes the demographic characteristics of the population.

**Table 1:** Demographic characteristics

Parameter	Value
Sex (F:M)	23:16
Mean age at consultation (years)	Mean: 44.5 (SD 19.1) Range: 5–80
Mean age at first episode (years)	Mean: 41.8 (SD 19.3) Range: 2–80
Time at follow-up (weeks)	Mean: 86.4 (SD 106) Range 0–341
Ethnicity	38 Hispanic 1 Caucasian

The clinical characteristics of the uveitis are shown in Table 2. Most patients had unilateral uveitis, followed by alternating bilateral and bilateral. All the patients had a non-granulomatous type of inflammation. The most common course was recurrent. The most prevalent anatomic diagnosis was anterior uveitis 76.9% (*n* = 30/39), followed by a combined presentation of anterior and intermediate uveitis that did not meet the criteria of panuveitis 15.4% (*n* = 6/39). Other clinical characteristics included initial BCVA with a mean of 0.179 LogMAR (SD 0.310 and range: 0–1.30), worst BCVA with a mean of 0.312 LogMAR (SD:0.616 and range: 0–3), anterior chamber cellularity with 0 + being the most common grading in the 47.4% (*n* = 32/38), followed by 0.5 + on 23.7% (*n* = 9/38). Most patients never presented anterior chamber flare, 82% (*n* = 32/38) on ophthalmologic examination, and all other flare gradings were evenly distributed between 0.5 + and 3 + with two patients each. Regarding vitreous cell count and haze, 50% (*n* = 18/36) and 67% (*n* = 25/39) had 0 + , respectively. Recurrences happened in 86.8% (*n* = 33) of the patients, with at least one during follow-up in 74.35% of the population studied.

**Table 2:** Clinical characteristics

**Parameter** (N)		**Value** n (%)
Laterality (*N* = 39)	Unilateral	*n* = 21 (53.8%)
	Alternating bilateral	*n* = 10 (25.64%)
	Bilateral	*n* = 8 (20.51%)
Onset (*N* = 39)	Insidious	*n* = 21 (53.8%)
	Sudden	*n* = 18 (46.2%)
Duration (*N* = 39)	Limited	*n* = 14 (35.9%)
	Persistent	*n* = 18 (46.2%)
Course (*N* = 39)	Acute	*n* = 4 (10.3%)
	Chronic	*n* = 9 (23.1%)
	Recurrent	*n* = 26 (66.7%)
Anatomic location (*N* = 39)	Anterior	*n* = 30 (76.9%)
	Anterior + Intermediate	*n* = 6 (15.4%)
	Intermediate	*n* = 1 (2.6%)
	Panuveitis	*n* = 2 (5.1%)
Initial BCVA (LogMAR)	Mean: 0.179 (SD 0.310) Range: 0–1.30	
Worst BCVA (LogMAR)	Mean: 0.312 SD:0.616 Range: 0.3	
Worst Cell Count (A.C.) (*N* = 38)	0 +	*n* = 18 (47.4%)
	0.5 +	*n* = 9 (23.7%)
	1 +	*n* = 4 (10.5%)
	2 +	*n* = 2 (5.3%)
	3 +	*n* = 1 (2.6%)
	4 +	*n* = 4 (10.5%)
Worst Flare (A.C.) (*N* = 38)	0	*n* = 32 (84.2%)
	0.5 +	*n* = 2 (5.3%)
	1 +	*n* = 2 (5.3%)
	2 +	*n* = 2 (5.3%)
	3 +	*n* = 2 (5.3%)
	4 +	*n* = 0
Worst Cell Count (Vitreous) (*N* = 36)	0 +	*n* = 18 (50%)
	0.5 +	*n* = 6 (16.7%)
	1 +	*n* = 7 (19.4%)
	2 +	*n* = 4 (11.1%)
	3 +	*n* = 1 (2.8%)
	4 +	*n* = 0
Worst Vitreous Haze (*N* = 39)	0	*n* = 25 (67.6%)
	0.5 +	*n* = 3 (8.1%)
	1 +	*n* = 3 (8.1%)
	2 +	*n* = 3 (8.1%)
	3 +	*n* = 1 (2.7%)
	4 +	*n* = 2 (5.4%)
Keratic precipitates	*n* = 10 (25.6%)	
Intraocular hypertension	*n* = 7 (17.9%)	
Intraocular hypotension	*n* = 5 (12.8%)	
Scleritis	*n* = 3 (7.7%)	
Hypopion	*n* = 1 (2.6%)	
Recurrences (n of recurrences during the follow up)	Mean: 1.8 Range: 0–4	
# Recurrences (*N* = 39)	0	*n* = 2 (5.7%)
	1	*n* = 13 (37.1%)
	2	*n* = 11 (31.4%)
	3	*n* = 8 (22.9%)
	4	*n* = 1 (2.9%)

*BCVA* Best Corrected Visual Acuity, *AC* Anterior Chamber

Other clinical characteristics of these patients included the presence of keratic precipitates in ten patients (25.6%), intraocular hypertension in seven (17.9%), hypotension in five (12.8%), scleritis in three (7.7%), anterior chamber (A.C.) fibrinous reaction in two (5.1%) and A.C. hypopyon in one (2.6%).

Most patients did not have systemic symptoms associated with uveitis (*n* = 26/39, 66.7%). However, eight (20.5%) had a diagnosis of ankylosing spondylitis (AS), two (5.1%) had reactive arthritis, rheumatoid arthritis, juvenile idiopathic arthritis, and TINU syndrome affected one patient each (2.6%). Additionally, regarding polyautoimmunity, seventeen had overt polyautoimmunity (47.2%), and four had latent polyautoimmunity (11.1%).

### Treatment and its impact on recurrences rate

The treatment strategy included all the therapeutic options shown in Table 3. The most used topical therapies were topical corticosteroids, non-steroidal anti-inflammatory drugs (NSAIDs), and the most common systemic therapies were methotrexate, oral corticosteroids, and a combination of methotrexate and adalimumab at the same time. Topical corticosteroids were used for 25.2 weeks on average (SD 39.7 and Range: 0–147) and systemic corticosteroids for 11.73 weeks (SD 34.8 and Range: 0–147).

**Table 3:** Treatments used in patients with HLA-B27-associated uveitis

**Systemic medications (*N* = 39)**	
Oral Corticosteroid	*n* = 8 (20.5%)
Intravenous Corticosteroid	*n* = 0
Intravitreal Corticosteroid	*n* = 0
Subconjunctival Corticosteroid	*n* = 5 (12.8%)
Methotrexate	*n* = 9 (23%)
Azathioprine	*n* = 1 (2.6%)
Sulfazalazine	*n* = 2 (5.1%)
Rituximab	*n* = 1 (2.6%)
Infliximab	*n* = 4 (10.3%)
Etanercept	*n* = 2 (5.1%)
Adalimumab	*n* = 5 (12.8%)
Certolizumab	*n* = 2 (5.1%)
Golimumab	*n* = 2 (5.1%)
MTX + ADA	*n* = 7 (17.9%)
**Topical medications (*N* = 39)**	
Corticosteroid	*n* = 30 (76.9%)
Hypotensor	*n* = 5 (12.8%)
Mydriatic	*n* = 14 (35.9%)
NSAIDs	*n* = 23 (59%)

*Legend*: Treatment strategies used in our patients, either for the management of the ocular or systemic disease. *MTX + ADA* Combined therapy of methotrexate and adalimumab, *NSAIDs* Non-steroidal anti-inflamatory drugs

The most common complications were cataracts and posterior synechiae, followed by macular edema, epiretinal membrane, band keratopathy, anterior synechiae, glaucoma, and papillitis. The frequency of each complication is shown in Table 4.

**Table 4:** Frequency of complications

Complications (*N* = 39)	
Cataract	*n* = 14 (35.9%)
Posterior synechiae	*n* = 13 (33.3%)
Macular edema	*n* = 6 (15.4%)
Epiretinal membrane	*n* = 5 (12.8%)
Band keratopathy	*n* = 3 (7.7%)
Anterior synechiae	*n* = 3 (7.7%)
Glaucoma	*n* = 2 (5.1%)
Papillitis	*n* = 1 (2.6%)

**Fig. 1:**
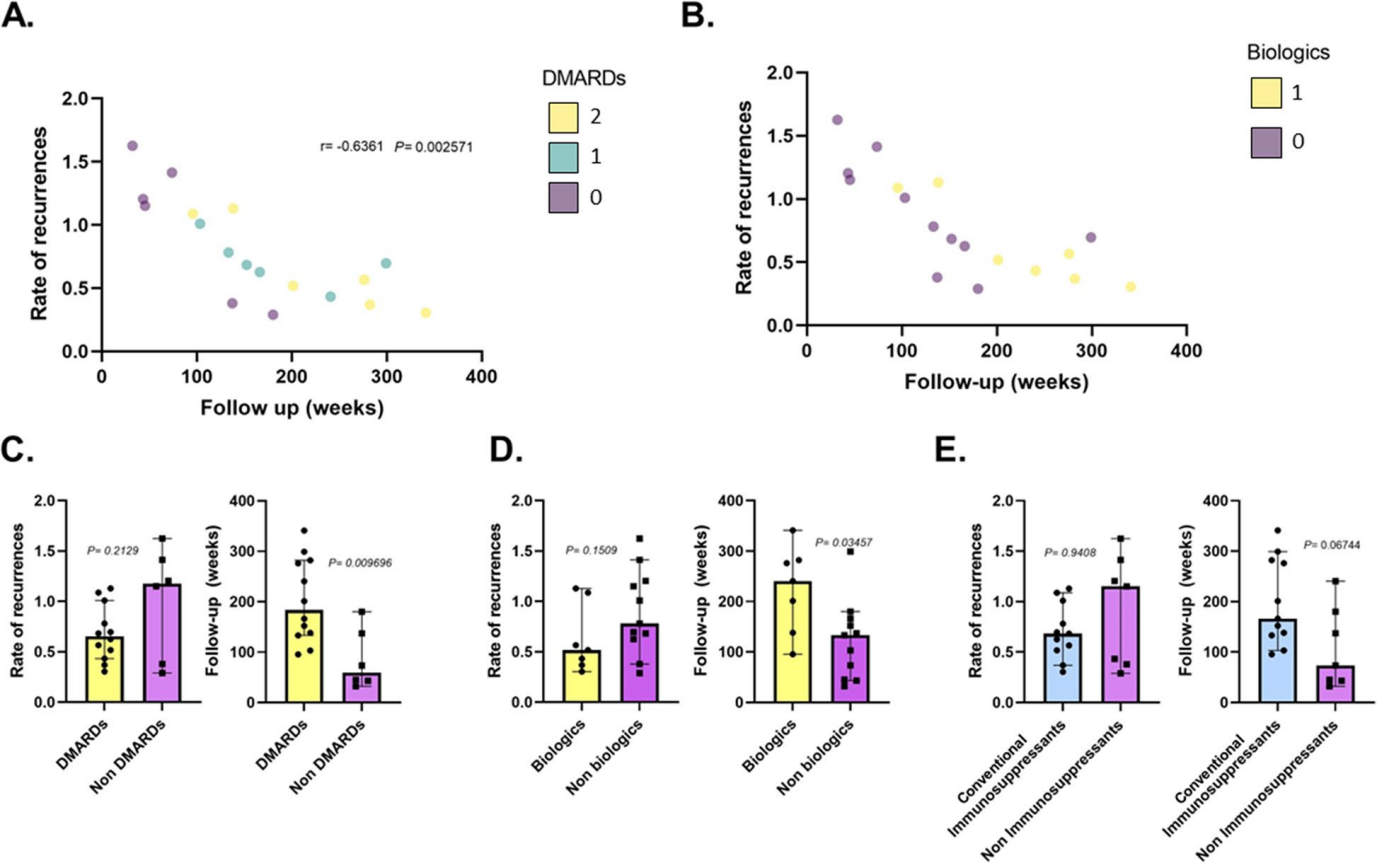
Evaluation of the impact of systemic treatment on the recurrence rate - Sub-analysis of patients with chronic and recurrent course Graph **A** shows the correlation between follow-up time and recurrence rate. It demonstrates that the recurrence rate diminishes as follow-up increases (Spearman's r = -0.6, *P* = 0.0). It also indicates that patients with 1 (green dots) and 2 (yellow dots) DMARDs present fewer recurrences over time. Graph **B** allows identification of which patients from Graph **A** received biologics (yellow dots) either as monotherapy or combined therapy. Graphs **C**, **D**, and **E** represent the difference in the mean recurrence rate and follow-up time between patients with and without therapy. The Mann–Whitney test was used in all cases. Graph **C** DMARDs Median "0.6549 recurrences per year," Non-DMARDs Median: "1.177 recurrences per year," *P* = 0.2129. DMARDs Median "183.6 weeks of follow-up," Non-DMARDs Median "59.40 weeks of follow-up," *P* = 0.0097. Graph **D** Biologic agents Median "0.5172 recurrences per year," Non-Biologic agents Median: "0.7814 recurrences per year," *P* = 0.1509. Biologic agents Median "240.6 weeks of follow-up," Non-Biologic agents Median "133.1 weeks of follow-up," *P* = 0.0346. Graph **E** Immunosuppressants Median "0.6833 recurrences per year," Non-Immunosuppressants Median: "1.150 recurrences per year," *P* = 0.4252. Immunosuppressants Median "166.0 weeks of follow-up," Non-Immunosuppressant agents Median "73.60 weeks of follow-up," *P* = 0.0441

On the other hand, the bivariate analysis between recurrences and medications, summarized in Table 5, showed no statistically significant results. Additionally, the recurrence rate was 1.5 and 1.42 during the first six and twelve months after the start of systemic medication. The sub-analysis on patients with chronic and recurrent course evidenced a negative correlation between follow-up time and rate of recurrences (*r* = -0.6361 *P* = 0.0025). However, the difference in the mean of recurrences between those with and without DMARDs did not achieve statistical significance (*P* = 0.2129), as the mean of follow-up did (*P* = 0.0096) (Fig. 1).

**Table 5:** Recurrences and medications

Medication	Frequency of use	*P-value*
Oral Corticosteroid	8/33 (24.2%)	*P* = 0.215
SC Corticosteroid	5/33 (15.2%)	*P* = 1
Methotrexate	9/33 (27.2%)	*P* = 0.181
Azathioprine	1/33 (3.0%)	*P* = 1
Sulfasalazine	2/33 (6.1%)	*P* = 1
Biologics	13/33 (39.4%)	*P* = 0.144
Rituximab	1/33 (3%)	*P* = 1
Infliximab	4/33 (12.1%)	*P* = 1
Etanercept	2/33 (6.1%)	*P* = 1
Adalimumab	4/33 (12.1%)	*P* = 0.527
Certolizumab	2/33 (6.1%)	*P* = 1
Golimumab	2/33 (6.1%)	*P* = 1
MTX + ADA	7/33 (21.2%)	*P* = 0.561

*Legend*: The number of patients who took each medication and had at least one recurrence during follow-up was expressed as a proportion (patients who took the medication and had a recurrence/total patients with recurrences) and percentage. A Fisher Exact test was performed to assess each association statistically. *SC* Subconjunctival, *MTX + ADA* Combined therapy of methotrexate and adalimumab

## Discussion

HLA-B27-associated uveitis is one of the most frequent specific causes of uveitis according to multiple studies [2, 3], representing 7.6% of uveitis in referral centers as was reported by McCannel et al. [3] which in turn closely resembles our findings (7.95%) and those reported by Abd el Latif et al. (7.1%) [18]. However, this data is significantly influenced by the study design and the prevalence of the HLA-B27 allele in the general population, reported as 8% in the Caucasian population from western countries and 1–5% in Africa, Asia, and Arabia [7]. Regarding sex distribution, previous studies conducted in Cuba [19], Egypt [18], and Colombia [11] reported a higher prevalence in males representing 56.5, 68.2 and 79.2% of cases, respectively, that contrasts with our findings since 59% of our patients were female. As for the age of onset, HLA-B27-associated uveitis patients are commonly described as young individuals between their 20' to 40' [7, 20]. Still, our findings demonstrate that in our population, the mean age of uveitis onset is slightly higher, being 41.8 years, and the disease onset could be at any age between 2 and 80 years.

In addition to demographic traits, our population's clinical spectrum of HLA-B27-associated uveitis slightly varied from the classical presentation described in the literature. Regarding laterality, an unusually high proportion of our patients presented simultaneous bilateral disease (20.58%) when compared to findings previously reported by several authors [7, 11, 18–23], including the Classification Criteria for Spondyloarthritis/HLA-B27-associated anterior uveitis by the SUN working group [4], who reported only 4% of patients with simultaneous bilateral disease. On the other hand, the anatomical diagnosis is often described as only anterior. Nonetheless, 17.9% of our patients had vitreous involvement, with 15.4% having a combined anterior and intermediate uveitis presentation; this strongly contrasts with the study done by Abd El Latif et al. [18], who reported vitreous involvement in only 6.5% of their patients, and Monnet et al. [23] who reported vitritis in 10.3% of their patients. In the context of recurrences, our findings are different from those reported by Power et al. [24], with an average number of recurrences of 3.6 (2–22) in contrast to our 1.5 and 1.42 mean of recurrences in six and twelve months.

Concerning BCVA, our findings showed that our patients had a significantly lesser visual impairment at presentation (0.179 LogMAR) when compared to studies conducted in Egypt [18] (0.66 LogMAR). We are more closely related to reports from Inanç [25] in Turkey, whose patients had a mean initial VA of 0.2 LogMAR. Given the design of our study, we did not report the final BCVA in our population since this may introduce uncontrolled bias and is not recommended by the SUN Guidelines for Reporting Clinical Data in Uveitis [5]. Therefore, we cannot compare this data with most published studies on this subject. We also found a notably lower than expected rate of A.C. fibrinous reaction and hypopyon (5.1 and 2.6%, respectively), considering that classical descriptions emphasize these characteristics as a hallmark of the disease. Even most prior studies reported rates ranging from 9.1 to 14.1% for hypopyon and 24.6 to 39.5% for A.C. fibrinous reaction [18, 22–24]. Only one study from China slightly resembled our findings, reporting rates of 6.8% for A.C. fibrinous reaction and 5.9% for hypopyon [26].

Multiple prior studies show a high percentage of patients with concurrent systemic disease among those with HLA-B27-associated uveitis, ranging between 38.8% and 77.7% [18, 19, 21–23, 25]. Our findings contrast with most of these studies since only a third (33.3%) of our patients were diagnosed with systemic disease; this could be explained due to differences in the follow-up period since uveitis can precede the onset of systemic disease by an average of eight years [18]. Moreover, our findings and those reported in the previously mentioned studies agree that ankylosing spondylitis is the most commonly-associated systemic condition in these patients. However, there is some degree of variation in the frequencies among the other systemic diseases, including other seronegative spondyloarthropathies [18, 19, 21–23, 25].

Additionally, we found that 11.1% of our patients presented latent polyautoimmunity, defined as the presence of several autoantibodies not directly related to the underlying autoimmune disease (A.D.) but with predictive value for an additional A.D [27]. Therefore, we suggest further research on the role of polyautoimmunity in uveitis, based on the fact that prior studies on other diseases have shown that combinations of different antibodies predict clinical variants of the disease [28] and may serve as a basis for the employment of secondary and tertiary preventive interventions in these patients to improve survival, morbidity, and quality of life [29].

HLA-B27-associated uveitis is typically managed with topical corticosteroids and mydriatic agents [9, 21]. However, given its recurrent nature, other therapeutic agents are used to avoid cumulative eye damage that increases the risk of vision impairment and blindness [10]. Several immunomodulatory therapies are used in uveitis, including methotrexate, azathioprine, mycophenolate, cyclosporine, and others, along with biologics[10]. More specifically, in HLA-B27-associated uveitis, Pascual et al. described sulfasalazine, methotrexate, adalimumab, and tocilizumab as the most used immunosuppressants to avoid recurrences or chronicity in ocular inflammatory activity not controlled with other treatments [30]. Additionally, Levy-Clarke et al. [31] proposed strong recommendations for infliximab or adalimumab as a corticosteroid-sparing treatment in patients with seronegative spondyloarthropathies and chronic uveitis. We found that methotrexate was the most used (23%) systemic treatment for patients with HLA-B27-associated uveitis, followed by oral corticosteroid (20.5%), and combination between combination methotrexate and adalimumab (17.9%).

Loh and Acharya [32] found in a retrospective longitudinal cohort study that the most common complications developed during follow-up in HLA-B27-associated uveitis are vision impairment of 20/50 (0.397 LogMAR) or worse (18%), posterior synechiae (17%), and cataract (14%). D'Ambrosio et al.[20] proposed that it is fundamental to have adequate control of the inflammation with topical steroids, a short course of systemic steroids, or topical NSAIDs to reduce the risk of complications and lead to a good visual outcome. In our cohort, despite the optimum treatment, the frequency of complication was slightly higher with a prevalence of cataract of 35.9%, posterior synechiae of 33.3%, and macular edema of 15.4%.

Generally, immunomodulators are initiated for the management of associated systemic disease, and sometimes it is necessary to initiate MTX specifically for the control of ocular flares. However, there are still no clinical guidelines with a high level of evidence about when to initiate immunomodulatory management to prevent recurrences in patients with HLA-B27-associated uveitis [33]. In our patients, we initiated DMARDs when more than two recurrences per year occurred in patients with isolated ocular involvement, or earlier if the rheumatologist deems it necessary in cases of associated systemic disease. Although the net recurrence rate was not different between those with and without DMARDs (*P* = 0.2129), those with DMARDs and biologics had longer follow-up (*P* = 0.009). Our analysis evidenced that the recurrence rate decreases as follow-up time progresses in patients with chronic and recurrent courses (*r* = -0.6361, *P* = 0.002571) (Fig. 1A), and the majority of patients with DMARDs were placed lower on the graph (green and yellow points in Fig. 1A). This allows us to infer that recurrence rates begin to decrease once DMARDs are initiated. However, prospective longitudinal studies are needed to quantify the real effect of DMARDs and the difference between conventional immunosuppressants and biologics.

Our study complements the few publications in the medical literature about the association between methotrexate (MTX) and relapses [9, 12, 13]. Zu Hoerste et al. [12] found that MTX treatment was associated with BCVA improvement and a decreased rate of cystoid macular edema, in addition to the effect of reduction of uveitis relapse rates in HLA-B27-positive AAU patients alongside sulfasalazine (SSZ). SSZ is also a good option with mild side effects and low cost, as suggested by Bouzid et al. [9], given its capability of reduction of recurrences with a Relative Risk (RR) of 85% and its effects on blood-aqueous barrier permeability during acute uveitis attacks [34]. More severely impaired patients or those with more severe systemic disease may benefit the most from biological therapy, including Golimumab [35, 36], Infliximab, Etanercept [37] and Adalimumab [38] as was suggested by our findings. TNFα inhibitors are excellent options since studies have demonstrated that TNFα inhibitor antibodies significantly reduce uveitis flares (RR 7.4) [39]. Moreover, patients under treatment with TNFα inhibitor antibodies have predominantly mild flares [40], and its efficacy in preventing relapses of rheumatological manifestations of spondyloarthropathies is strongly evidenced [41, 42].

The limitations of this study include its retrospective nature, a small sample, and referral bias. Given that most patients with HLA-B27-associated uveitis may be diagnosed and treated in a general ophthalmology practice, one could argue our sample represents a minority in which diagnosis or treatment was difficult. Furthermore, previous studies in Colombia reveal a more significant delay of referral to the uveitis specialist in comparison to other countries[43], with a mean of 2.08 years between the appearance of uveitis symptoms and the uveitis specialist's first evaluation, with anterior uveitis having the more significant delay in referral, thereby reducing the representativeness of our sample.

## Conclusion

The clinical spectrum of HLA-B27-associated uveitis in Colombian patients differs from other studies conducted on different latitudes, notably female predominance, older age at presentation, higher frequency of bilateral and vitreous involvement, and lower frequency of concomitant systemic diseases. Additionally, our results suggest that DMARDs such as methotrexate and biologic agents are good therapeutic options to avoid recurrences in chronic and recurrent cases.

## Data Availability

The information in the databases used in this article is freely accessible and available for research purposes from the corresponding author on reasonable request.

## Abbreviations

AAU: Acute anterior uveitis
AC: Anterior chamber
CRP: C-reactive protein
CYC: Cyclosporine
FTA-ABS: Fluorescent treponemal antibody absorption
HLA-B27: Human Leukocyte Antigen B27
IV: Intravenous
MTX + ADA: Combined therapy of methotrexate and adalimumab
NSAIDs: Non-steroidal Anti-Inflammatory Drugs
P.O: Per Os (Oral administration)
RR: Relative Risk
SC: Sub-Conjunctival
SD: Standard deviation
SSZ: Sulfasalazine
SUN: Standardization of Uveitis Nomenclature
TINU: Tubulointerstitial Nephritis and Uveitis Syndrome
TNFa: Tumor Necrosis Factor-alpha
VA: Visual Acuity
VDRL: Venereal disease research laboratory

## References

[1] Tsirouki T, Dastiridou A, Symeonidis C, Tounakaki O, Brazitikou I, Kalogeropoulos C, Androudi S (2018). A focus on the epidemiology of uveitis. Ocul Immunol Inflamm.

[2] Jakob E, Reuland MS, Mackensen F, Harsch N, Fleckenstein M, Lorenz H-M, Max R, Becker MD (2009). Uveitis subtypes in a German interdisciplinary uveitis center—analysis of 1916 patients. J Rheumatol.

[3] McCANNEL CA, Holland GN, Helm CJ, Cornell PJ, Winston JV, Rimmer TG, THE UCLA COMMUNITY-BASED UVEITIS STUDY GROUP (1996). Causes of uveitis in the general practice of ophthalmology. Am J Ophthalmol.

[4] (2021) Classification Criteria for Spondyloarthritis/HLA-B27-Associated Anterior Uveitis. Am J Ophthalmol 228:117–125. 10.1016/j.ajo.2021.03.049 10.1016/j.ajo.2021.03.049 PMC8594762 33845004

[5] (2005) Standardization of uveitis nomenclature for reporting clinical data. Results of the first international workshop. Am J Ophthalmol 140:509–516. 10.1016/j.ajo.2005.03.057 10.1016/j.ajo.2005.03.057 PMC8935739 16196117

[6] de-la-Torre A, López-Castillo CA, Rueda JC, Mantilla RD, Gómez-Marín JE, Anaya J-M (2009) Clinical patterns of uveitis in two ophthalmology centres in Bogota, Colombia.Clin Exp Ophthalmol 37:458–466. 10.1111/j.1442-9071.2009.02082.x 10.1111/j.1442-9071.2009.02082.x 19624341

[7] Pathanapitoon K, Dodds EM, Cunningham ET, Rothova A (2017). Clinical spectrum of HLA-B27-associated ocular inflammation. Ocul Immunol Inflamm.

[8] Wakefield D, Chang JH, Amjadi S, Maconochie Z, el-Asrar AA, McCluskey P (2011). What Is New HLA-B27 Acute Anterior Uveitis?. Ocul Immunol Inflamm.

[9] Bouzid N, Jamilloux Y, Chapurlat R, Pradat P, De Parisot A, Kodjikian L, Sèves P (2020). Impact of systemic treatments on the course of HLA-B27-associated uveitis: a retrospective study of 101 patients. PLOS One.

[10] Dick AD, Rosenbaum JT, Al-Dhibi HA, Belfort R, Brézin AP, Chee SP, Davis JL, Ramanan AV, Sonoda K-H, Carreño E, Nascimento H, Salah S, Salek S, Siak J, Steeples L, Accorinti M, Acharya N, Adan A, Agrawal R, Akkoc N, Al Ghamdi S, Al Ghamdi T, Al Saati A, Alsabaani N, Al-Shamarani M, Bachta A, Barisani-Asenbauer T, Beare N, Porto FBO, Blanco R, Yee ACS, Chandran V, Chiquet C, Chng HH, Cimbalas A, Cimino L, Cordero-Coma M, Cristobal C, Cuevas M, Eurico da Fonseca J, de Boer J, de la Torre A, De Schryver I, Derzko-Dzulynsky L, Diaz-Valle D, Merino CED, Facsko A, Figueira L, Fonollosa A, Fortin E, Gale R, Galeazzi M, Garcia S, Ruiz G, de Morales JM, Gašperšič N, Goldstein D, Guedes M, Guex-Crosier Y, Gul A, Hamam R, Haroon M, Hasegawa K, Heiligenhaus A, Hooper C, Hwang Y-S, Hwang D-K, Juanola X, Kaburaki T, Kadayifcilar S, Kempen J, Kezuka T, Kherani A, Kirsimäe M, Kotaniemi K, Kraut A, Kubicka-Trząska A, Kuffova L, Lightman S, Lim L, Lim WK, McCluskey P, McGuire M, Mirabelli P, Miserocchi E, Misiuk-Hojło M, Muccioli C, Muñoz S, Murphy C, Murray PI, Nagy Z, Namba K, Neri P, Nguyen Q, O’Gradaigh D, Omair M, Otsa K, Ozyazgan Y, Pablo F, Paroli MP, Pleyer U, Poór G, Proença R, Rabinovitch T, Read R, Rebrov M, Recillas-Gispert C, Rothova A, Schwartzman S, Seve P, Sharma S, Sims J, Sohár N, Suhler E, Szántó S, Szepessy Z, Tappeiner C, Thorne J, Tugal Tutkun I, Turno-Kręcicka A, Van Calster J, van der Horst-Bruinsma I, Vidovič Valentinčič N, Vitale A, Voorduin Ramos S, Vukojevic N, Wakefield D, Willermain F, Yalcindag N, Yamamoto JH, Yeh S, Zemaitiene R, Ziouzina O (2018). Guidance on noncorticosteroid systemic immunomodulatory therapy in noninfectious uveitis. Ophthalmology.

[11] Molina CA, Donado JH, Vélez LM, et al (2007) Uveitis en pacientes con espondiloartropatías seronegativas. Hospital Pablo Tobón Uribe, Medellín, Colombia. Colomb Médica 38:382–385. http://www.scielo.org.co/scielo.php?script=sci_arttext&pid=S1657-95342007000400007

[12] zu Hoerste MM, Walscheid K, Tappeiner C, Zurek-Imhoff B, Heinz C, Heiligenhaus A,  (2018). The effect of methotrexate and sulfasalazine on the course of HLA-B27-positive anterior uveitis: results from a retrospective cohort study. Graefes Arch Clin Exp Ophthalmol.

[13] Muñoz-Fernández S, García-Aparicio AM, Hidalgo MV, Platero M, Schlincker A, Bascones ML, Pombo M, Morente P, Sanpedro J, Martín-Mola E (2009). Methotrexate: an option for preventing the recurrence of acute anterior uveitis. Eye.

[14] von Elm E, Altman DG, Egger M, Pocock SJ, Gøtzsche PC, Vandenbroucke JP, STROBE Initiative (2007). The Strengthening the Reporting of Observational Studies in Epidemiology (STROBE) statement: guidelines for reporting observational studies. PLoS Med.

[15] Armstrong RA (2013). Statistical guidelines for the analysis of data obtained from one or both eyes. Ophthalmic Physiol Opt.

[16] Schulze-Bonsel K, Feltgen N, Burau H, Hansen L, Bach M (2006). Visual acuities “Hand motion” and “Counting fingers” can be quantified with the Freiburg visual acuity test. Investig Opthalmology Vis Sci.

[17] Moussa G, Bassilious K, Mathews N (2021) A novel excel sheet conversion tool from Snellen fraction to LogMAR including ‘counting fingers’, ‘hand movement’, ‘light perception’ and ‘no light perception’ and focused review of literature of low visual acuity reference values. Acta Ophthalmol (Copenh) 99. 10.1111/aos.14659 10.1111/aos.14659 33326177

[18] Abd El Latif E, Abdelhalim AS (2020). Clinical profile of HLA-B27-associated uveitis in an Egyptian cohort. Clin Ophthalmol.

[19] Torres S, Borges S, Artiles A (2013). HLA-B27 and clinical features of acute anterior uveitis in Cuba. Ocul Immunol Inflamm.

[20] D’Ambrosio EM, La Cava M, Tortorella P, Gharbiya M, Campanella M, Iannetti L (2017). Clinical features and complications of the HLA-B27-associated acute anterior uveitis: a metanalysis. Semin Ophthalmol.

[21] Wakefield D, Easter J, Penny R (1984). Clinical features of HLA-B27 anterior uveitis. Aust N Z J Ophthalmol.

[22] Tay-Kearney M-L, Schwam BL, Lowder C, Dunn JP, Meisler DM, Vitale S, Jabs DA (1996). Clinical features and associated systemic diseases of HLA-B27 uveitis. Am J Ophthalmol.

[23] Monnet D (2004). Ophthalmic findings and frequency of extraocular manifestations in patients with HLA-B27 uveitis*1A study of 175 cases. Ophthalmology.

[24] Power W (1998). Outcomes in anterior uveitis associated with the HLA-B27 haplotype. Ophthalmology.

[25] İnanç M, Şimşek M, Çakar Özdal MP (2019). Etiological and clinical characteristics of HLA-B27-associated uveitis in a tertiary referral center. Turk J Ophthalmol.

[26] Yang P, Wan W, Du L, Zhou Q, Qi J, Liang L, Wang C, Wu L, Kijlstra A (2018). Clinical features of HLA-B27-positive acute anterior uveitis with or without ankylosing spondylitis in a Chinese cohort. Br J Ophthalmol.

[27] Molano-González N, Rojas M, Monsalve DM, Pacheco Y, Acosta-Ampudia Y, Rodríguez Y, Rodríguez-Jimenez M, Ramírez-Santana C, Anaya J-M (2019). Cluster analysis of autoimmune rheumatic diseases based on autoantibodies. New insights for polyautoimmunity. J Autoimmun.

[28] Meroni PL, Borghi MO (2018). Diagnostic laboratory tests for systemic autoimmune rheumatic diseases: unmet needs towards harmonization. Clin Chem Lab Med CCLM.

[29] Tobón GJ, Pers J-O, Cañas CA, Rojas-Villarraga A, Youinou P, Anaya J-M (2012). Are autoimmune diseases predictable?. Autoimmun Rev.

[30] Valls Pascual E, Fontanilla Ortega P, Vicens Bernabeu E, Martínez-Costa L, Blanco Alonso R (2016). Características clínicas, tratamiento y complicaciones oculares de uveítis anterior asociada y no asociada a HLA-B27. Reumatol Clínica.

[31] Levy-Clarke G, Jabs DA, Read RW, Rosenbaum JT, Vitale A, Van Gelder RN (2014). Expert panel recommendations for the use of anti-tumor necrosis factor biologic agents in patients with ocular inflammatory disorders. Ophthalmology.

[32] Loh AR, Acharya NR (2010). Incidence rates and risk factors for ocular complications and vision loss in HLA-B27-associated uveitis. Am J Ophthalmol.

[33] Chao YJ, Hung JH, Lin CP, Kuo HK, Chen SN, Hwang YS, Li KJ, Lin CJ, Hwang DK, Sheu SJ (2023) Diagnosis, treatment, and prevention of noninfectious acute anterior uveitis with or without human leukocyte antigen B27 in adults - expert consensus in Taiwan. Ocul Immunol Inflamm 1–8. 10.1080/09273948.2023.2165113 10.1080/09273948.2023.2165113 36701640

[34] Benitez-Del-Castillo JM, Garcia-Sanchez J, Iradier T, Bañares A (2000). Sulfasalazine in the prevention of anterior uveitis associated with ankylosing spondylitis. Eye.

[35] Yazgan S, Celik U, Işık M, Yeşil NK, Baki AE, Şahin H, Gencer E, Doğan İ (2017). Efficacy of golimumab on recurrent uveitis in HLA-B27-positive ankylosing spondylitis. Int Ophthalmol.

[36] Calvo-Río V, Blanco R, Santos-Gómez M, Rubio-Romero E, Cordero-Coma M, Gallego-Flores A, Veroz R, Torre I, Hernández FF, Atanes A, Loricera J, González-Vela MC, Palmou N, Hernández JL, González-Gay MA (2016). Golimumab in refractory uveitis related to spondyloarthritis. Multicenter study of 15 patients. Semin Arthritis Rheum.

[37] Braun J, Baraliakos X, Listing J, Sieper J (2005). Decreased incidence of anterior uveitis in patients with ankylosing spondylitis treated with the anti-tumor necrosis factor agents infliximab and etanercept. Arthritis Rheum.

[38] Kim M, Won J-Y, Choi SY, Ju JH, Park Y-H (2016). Anti-TNFα treatment for HLA-B27-positive ankylosing spondylitis-related uveitis. Am J Ophthalmol.

[39] Guignard S, Gossec L, Salliot C, Ruyssen-Witrand A, Luc M, Duclos M, Dougados M (2006). Efficacy of tumour necrosis factor blockers in reducing uveitis flares in patients with spondylarthropathy: a retrospective study. Ann Rheum Dis.

[40] Rudwaleit M, Rødevand E, Holck P, Vanhoof J, Kron M, Kary S, Kupper H (2009). Adalimumab effectively reduces the rate of anterior uveitis flares in patients with active ankylosing spondylitis: results of a prospective open-label study. Ann Rheum Dis.

[41] Braun J, de Keyser F, Brandt J, Mielants H, Sieper J, Veys E (2001). New treatment options in spondyloarthropathies: increasing evidence for significant efficacy of anti–tumor necrosis factor therapy. Curr Opin Rheumatol.

[42] Braun J, Brandt J, Listing J, Zink A, Alten R, Golder W, Gromnica-lhle E, Kellner H, Krause A, Schneider M, Sörensen H, Zeidler H, Thriene W, Sieper J (2002). Treatment of active ankylosing spondylitis with infliximab: a randomised controlled multicentre trial. The Lancet.

[43] Villalobos-Pérez A, Reyes-Guanes J, Muñoz-Ortiz J, Estévez-Florez MA, Ramos-Santodomingo M, Balaguera-Orjuela V, de-la-Torre A,  (2021). Referral process in patients with uveitis: a challenge in the health system. Clin Ophthalmol.

